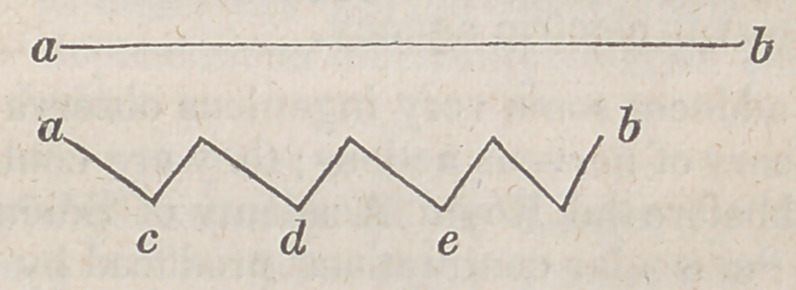# Analectic

**Published:** 1833

**Authors:** 


ANALECTIC, ANALYTICAL, AND ORIGINAL.

### I. Analectic.

### *Ad Sylvas Nuncius.*

1. *Observations on Cold, as a Cause of Disease.* By Dr. *J. Clendenning.*—In our valued and oldest contemporary, the Med. and Phys. Journal, Dr. Clendinning has published a monograph on that popular and real cause of multiplied evils—COLD. Every medical practitioner is aware that nine-tenths of the diseases presented to his observation, are attributed by the sufferers to catching—"COLD"—and there must be some, nay, there must be much foundation in truth for so general a persuasion. From the cold wash of our first nurse, to the heats and chills of our juvenile sports, and unavoidable exertions of our riper years, the effects of cold, or rather of atmospherical transitions, thermometrical and hygrometrical, are daily conspicuous to the common as well as the medical observer. It has been recorded by Dr. Bateman that, during the winter of 1814, which was very severe, the number of patients at the Cary Street Dispensary exceeded by 700 the ordinary average in other years! Dr. Heberden also records the fact that, in January, 1795, the whole mortality of London, was *double* that of the succeeding January. We question if the redoubted cholera of January, 1832, has produced such a tremendous change in the balance of our final accounts with grim Death.

The author of this monograph, a gentleman of highly cultivated mind and excellent education, general as well as professional, has arranged his observations under six heads—definition of terms—morbific properties of cold—diseases of cold—principal forms of morbific cold—circumstances most favorable to the morbific action of cold—and lastly, the means of preventing diseases of cold. These subjects, so clearly arranged, are scientifically treated, and ingeniously illustrated by Dr. Clendinning. We regret that, from the terse and didactic manner in which the talented author discusses each point, we are quite unable to attempt an analysis of the paper. We are therefore induced to insulate one or two propositions and give them in the writer's own language, as they will prove interesting in themselves, and afford a fair specimen of the whole performance, which we urgently recommend to the attention of our readers.

"FORMS OF COLD MOST DANGEROUS.

The principal and most active forms of morbific cold met with in practical life are three: *moist atmospheres, damp clothing*, and *currents of air*.

Moisture of itself is not injurious to health. Moist warm atmospheres are indifferent to the vigorous, and they are generally favorable to the weakly. *Wet* summers are healthful in this country, provided they are not cold; the summer of 1797 furnishes a very striking proof of this truth. 'From the middle of May it was,' says HEBERDEN, 'one of the wettest ever remembered, it was nevertheless in every respect a healthy year.' Not so, however, *wet cold* seasons. BATEMAN assures us 'that the succession of rains to heat' (i. e. of a cool or cold moisture to warmth,) 'is amongst the most active causes of disease of the chest and abdomen,' which are the most destructive complaints in this metropolis. 'A foggy atmosphere,' he again observes,' acts much more injuriously than a clear (i. e. comparatively dry) one of equal cold. Indeed there is,' he assures us, 'no condition of the air so invariably pernicious, so chilling and oppressive to the organs of respiration, as that frequent combination of frost with fog in the metropolis.' Of the truth of the preceding observations of that judicious physician (Bateman,) I have had frequent experience amongst the poor inhabitants of Westminster for the last three years, during which I have had considerable opportunities of watching the operation of weather and season.

The danger of inhabiting or sleeping in *damp apartments*, is proved by examples of daily occurrence in private life. The superior morbific activity of a *damp atmosphere* depends on its superior conductive power. A humid air absorbs free caloric with much greater avidity and rapidity than a dry.

*Damp clothing* is another active and dangerous form of cold. The mischievous energy of wet clothes is so well known, as to require no illustration. The great capacity of evaporating water for the matter of heat, is the cause. The frigorific power of damp clothing may be conceived from this consideration; that the only protection or antagonist influence that man requires to enable him to defy the summer fires of Sahara or South Carolina, is the power of cutaneous exhalation. Although inhaling and immersed for sometime in an atmosphere exceeding very far the temperature of boiling water, the bakers' girls were found, by Reaumur, to have pertinaciously retained their normal heat. After twelve or fifteen minutes' immersion in an atmosphere many degrees above 212°, BLAGDEN, BANKES, DOBSON, and other experimenters, found their thermometrical heat little differing from that of ordinary health. Such is the frigorific power of perspiration, or, in other words, of evaporation from the surface.

But *currents of air*, perhaps, of all causes of diseases of cold, are the most active and extensively mischievous. *Damp clothes* may be avoided; *foggy atmospheres*, and extremely *humid cold winds*, are unknown in many seasons and climates: but *currents of air* must be encountered. The atmosphere is constantly in a state of agitation; its intestine and progressive motions, while, on the one hand, they promote our well-being by ventilation, endanger, on the other hand our health and our existence by their refrigerant operation. The destructive power of exposure to cold winds without adequate protection, is strikingly illustrated by the narrative published by Dr. CURRIE, in the Philosophical Transactions for 1792. 'Of several individuals that clung to the wreck, two sat on the only part that was not submerged; of the others all were constantly immersed in the sea, most up to the shoulders; three only perished, two of whom were generally out of the sea, but frequently overwhelmed by the surge, and at other times exposed to heavy showers of sleet and snow, and to a high and piercing wind.' Of these two, one died, after four hours' exposure; the second died three hours later, 'although a strong healthy man of twenty-eight, a native of Scotland, in the flower of life, early inured to cold and hardship, and very vigorous both in mind and body.' The third that perished had been a weakly man. The remaining eleven who had been more or less completely *submerged*, were taken from the wreck next day, after 23 hours' exposure, and recovered. The person amongst the whole who seemed to have suffered least was a negro: of the other survivors, several were by no means strong men, most of them had been inured to the warm climate of Carolina.' In the case of the first two that perished the morbific power of the 'high piercing wind' was aided no doubt very powerfully by evaporation. In Dr. Currie's account of his experiments on the cold bath. we have the following interesting illustration of the superior refrigerant power of wind or air in motion. After continuing in the water fifteen minutes, the subject of some of his trials exhibited 'little or no diminution of his heat in rising into the air in a perfect calm, though during a frost; while the like exposure in a second trial, under similar circumstances, but with a north-east wind blowing sharply, produced a rapid diminution (of animal heat,) though the air was many degrees warmer' than in the preceding experiment.

I have above cited several examples of even death instantaneously produced by the chilling influence of a piercing north wind. Every valetudinarian is aware of the inconvenience and even danger of exposure to blasts from chinks and other apertures in rooms otherwise close.

### *Prevention of Diseases of Cold.*

The remarks I have to make in this section will come under the head of *Clothing*, *Exercise*, *Internal heat*, or stimulating ingesta, and *Diaphoretic Means*, as hot diluents, bed-heat, &c.

Every considerable augmentation of refrigerant influence requires on the part of the subject exposed, proportionate precautionary means for the protection of health: these preventive measures must consist either of increased clothing or of the use of means capable of compensating for defect of personal coverings, by diminution of intrinsic organic susceptibility. The class of preventive means last alluded to, will be by and by considered, under the heads of exercise and stimulating ingesta: at present I shall confine myself to the question of clothing.

Transition from a tranquil into an agitated or progressive atmosphere, as from indoors into the open air, from the inside of a stage coach to the outside, &c., is accompanied with a great increase of the refrigerant power, which the frame has to encounter, and will, in many instances, above all if moisture be present, require additional protective covering. When the exposure is but short, or the weather is fine, or the constitution vigorous, and reactive energy therefore ample, such precaution, no doubt, will generally be quite unnecessary: yet those compensative conditions must be often wanting in a greater or less degree, and exposure, therefore, not provided against by appropriate internal or external means, will often prove hazardous, and sometimes fatal. In how many cases has phthisis been traced to an indiscretion of the sort now alluded to; to a journey on the top of a stage-coach in bad weather, or by night with insufficient clothing, &c. In how many instances have youth and accomplishment, and loveliness, fallen victims to the noxious influence of cool, perhaps damp out-of-doors atmospheres in passing from one rout to another, or returning from scenes of splendid riot to domestic solitude and repose.

In passing from a state of activity or exertion to one of relative quietude, precautions are often required for security; such transitions occur when horse or foot exercise is exchanged for riding in an open carriage, or gestation on the water, and obviously demand the like precautions with transitions from walking, running, &c., to sitting, lying down, &c. But of all conditions that require provident measures, that of sleep stands most in need of them. In that condition the calorific function is less excited, less exposed to incidental stimulation from physical agents, or moral impulses, or muscular exertion, than in any other. Less heat is evolved; the body is much more readily chilled, the cutaneous functions more easily disturbed, and every derangement of internal parts, producible by frigorific impressions on the skin is more promptly effected. In the state of sleep, it is therefore, if ever, necessary to guard against exposure to cool moisture, currents of cool air, and every other cause of diseases of cold. All this is very plain, and is generally known, and requires no further notice. Before quitting this topic, however, I would briefly enter my protest against the absurd and mischievous extreme to which many, perhaps most people, carry the use of woollen and other night clothing. It is common for females, in particular, who seldom, amongst the richer classes at least, know the comfort, the real luxury of woollen or chamois coverings for the shoulders, chest, feet, &c., and who wear below the knee, on the arms, upper part of the chest, neck, or head, either slight or no covering, to retire to sleep on beds of feathers, under half a dozen or more folds of one material or another, mostly woollen, and this in soft, nay even in summer weather, and with every avenue for fresh air, every door and window, closed, and bed-curtains perhaps drawn closely all around: from such violent transitions what wonder if inconvenience result! The sleep is more or less disturbed by dreams and feverish uneasiness; the strength is not properly recruited, and the sleeper awakes unnerved, languid, indolent, often hot or chilly, generally anorectic. Under such circumstances, a susceptibility of inconvenience and injury from cold, above the average, may reasonably be looked for, and will, I believe, seldom fail, if occasion offer, to show itself. Nor is the relaxation attending long immersion in warm air the only disadvantage in such cases; for there is obviously the further one of long-continued respiration of an impure atmosphere to be taken into the account: a disadvantage of no trifling importance in the cases of such as retire early to small rooms, and emerge into daylight after protracted slumbers.

Another point in which many fail, is the adaptation of clothing to season, weather, &c. No one questions the propriety of such adaptation in the abstract; but the number of those that commit the grossest errors on this subject in practice is enormous. What can be more obvious than the temerity of wearing the same kind and quantity of clothing in the heats of summer and frosts of winter; yet there are not wanting in the very first rank of the medical profession persons chargeable with such imprudence. I recollect very well the substance of an argument I once had with a fellow-traveller, an Austrian cadet, on his way through mountains in mid-winter, *en voiture*, from Vienna to Laybach. He obviously suffered inconvenience from want of warmer clothing, yet would not admit the propriety of adding even a flannel vest to his wardrobe. He considered it, he told me, 'militarisch,' soldier-like, to dispense with woollen under-coverings. A like answer would no doubt be given by many defaulters on this side of the water. Ladies would hold it to be *feminine*, gentlemen, *manly*, &c., to dispense with the extra under-clothing proper for winter and cold weather. But indolence, temerity, and fine breeding, are bad protectives against inclement seasons.

*Exercise.* An observant individual can seldom fail to know when, from universal weakness or incidental exposure, he is in danger from external cold; and a provident man will easily, in general, foresee future exposure. When actually exposed, the great prophylactic is muscular exertion, and, if possible, locomotive exercise. Ritter's advice is excellent, when he recommends that we should counteract the chilling influence of a draught or of a damp atmosphere, to which we are constrained to expose ourselves, by proportionably increased exercise in order that we may be enabled to compensate for the augmented expenditure of caloric by an increased evolution of it. The calorific power of general muscular exertion is such that, but for the antagonist frigorific power of cutaneous exhalation and evaporization, there can be no doubt that even moderate exercise would be incompatible with health, and that violent locomotive exertion would, in comparatively tranquil atmospheres at least, prove destructive of life. It is so great, that, daily persevered in, and aided by clothing sufficient to protect the skin and extremities from the immediate contact of an intensely cold air, it has been, on innumerable occasions, found sufficent to bear man harmless through the most formidable trials, as the narratives of Parry, Franklin, Scoresby, and many others, abundantly testify.

Respecting the use of *hot drinks* and *aliments at once nutritive and stimulant*, before and during exposure, little need be said. All experience is in their favor; every traveller on our stage-coaches knows the protecting power of warm tea and coffee, punch, &c.; there is even unequivocal experimental proof of the power of stimulant drinks to sustain the animal temperature under exposure. During my experiments on the cold bath, I found, in some trials with warm drinks and wine, (taken before immersion,) the sensation of cold little less lively indeed, and the access of shivering little retarded; but the pulse and heat under the tongue were much less reduced by the cold than in other trials made without such preparation. As a preparative, however, for protracted exposure to cold, &c., pure vinous liquors are obviously unsuitable means: the excitement they produce is transitory, and is followed by dangerous depression of calorific power: and their repeated and free use is, amongst other objections, liable to this, that it favors that somnolency which is one of the most perilous effects of cold. I have little doubt that the protective power of punch, negus, &c. is more owing to the hot water than to the pungent spirit.

The fourth division comprises the means of cutting short incipient diseases of cold. On the supervention of chillness and other symptoms, effects of recent exposure to cold, such as slight headach, horripilation, dejection of spirits, hoarseness, slight sore throat, coryza, lachrymation, cold feet, anorexia, lumbar pains &c., we should have immediate recourse to the shelter of a warm bed; all solid aliment should be withheld; our only ingesta should be warm diaphoretic drinks. Diluted vinous liquors taken warm, such as weak hot punch or negus, are often excellent diaphoretics in such cases. But, in general, the alcoholic ingredients may be safely dispensed with, and when the excitement is considerable and headach is present, it cannot, without rashness, be recommended. The preceding measures are usually sufficient, if early enough employed, to cut short incipient derangements from cold. Where irritation is considerable, which is indicated by flying pains in the back and limbs, lively sense of cold, smart shivering, &c., opiates had better be employed in addition to the means already mentioned: for this purpose Ritter highly extols a combination of opium and camphor, two or four grains of the latter with from the eighth to the fourth part of a grain of the former every second hour, until the horrors, headach, pains, &c., shall have vanished or greatly declined. I have no doubt of the utility of such a combination; but pure laudanum or opium combined with warm diluents will probably be found fully as efficient. Dover's powder is also an excellent remedy. Another remedy, at once efficient and agreeable, is the common effervescing draught, containing half a scruple of nitre, a drachm (more or less) of the compound tincture of camphor, and in some cases half a drachm or more of nitrous æther, and as much of Hippo wine, to be repeated every third, fourth, or sixth hour. Where the feeling of cold, as evidenced by horripilation, rigors, &c. is lively, warm bathing, local or general, followed up by some of the remedies just proposed, is very proper.

Prevention of disease is better than cure; it implies a more masterly degree of skill and power in the prescriber, and a smaller expense of care and vital power on the part of the sick. In practical medicine of the first indication in dignity as well as time, is prevention: in other words, the avoidance or counteraction, as far as possible, of morbific agencies; and when illness arrives, the employment, without loss of time, of the means best calculated to disperse the earlier groups of organic preternatural conditions or symptoms, and thus, by anticipation, get rid of the complications and difficulties so soon superinduced and accumulated upon primary simple and tractable derangements by the influence of sympathy and habit: with these views, I have thought it advisable to append to my observations on the morbid effects of cold, remarks on the circumstances that most favor the action of morbific cold, on the means best calculated to neutralize its agency, and on the remedies that should be employed after injurious exposure to prevent the establishment of any nosological effect or regular disease of cold: on the plan, as on the execution, it is the reader's province to decide."

The whole monograph which would have well deserved a place in the Cyclopædia of Practical Medicine, or in Dr. Copland's Dictionary, contains the most convincing proofs of the author's learning, talents, and discrimination.

2. *On Muscular Contractions, and Animal Electricity.*—The change which takes place in a muscular fibre at the moment of contraction, consists in its assuming a zig-zag flexuosity, and is best illustrated by the following diagram, which at the same time points out the amount of shortening.

This may be stated to be nearly one-fourth, or 0.23. The above appearance is most readily detected by submitting a very delicate muscle, as the sterno-pubic of a frog, to a microscope; and at the same time we may observe, that the summits of the angles, *c*, *d*, *e*, correspond precisely to the junction of the minute nervous filaments, which, when the muscle is relaxed, run parallel to each other, and perpendicular to the muscular fibres. Admitting the correctness of this opinion, it will easily be conceived that the living muscle is really a galvanometer, and one too of extraordinary sensibility. In all cases, where muscular contractions are produced, there also exists a developement of electricity. For the purpose of displaying this, let two similar platina wires be fitted to the ends of the branches of a galvanometer; let one of them be plunged into the muscles of a frog, and let the nerves of the animal be touched with the other, heated to redness; the contractions will be strong, and the deviation of the needle very sensible.

We have ascertained by experiment, that when two living animal substances are pressed together, however slightly, they acquire opposite states of electricity. It is sufficient for two insulated persons to touch hands, and then withdraw from the contact; to develope electricity sufficient to affect the electroscope of Caulomb.

The insulation of the nervous fibres is effected by the abundant fatty matter which surrounds them—for the discovery of this we are indebted to Vauquelin, who has shewn that it envelopes each of the fi res, and does not permit the electric fluid to pass from one to the other.

It is a subject of curious investigation to try to explain how some secretions, as the milk, chyle, urine, and the sweat are acid; while others, as the saliva, bile, &c., are strongly alkaline. The fluid from which they are all derived, namely, the blood, contains pure caustic soda, in sufficient quantity to impart to it manifest alkaline properties. If we seek among the facts of chemistry for an explanation of this difference between the constitution of the blood, and that of the fluids secreted from it, we may soon be convinced that the action of the voltaic pile is the only one which approaches to it. Moreover it appears possible, to imitate artificially the principal conditions of the secretions, and to separate from the blood by means of the pile, a liquid resembling milk, and from the food itself, a material resembling chyle. We cannot quit this most interesting subject without remarking, that if muscular motion and the secretions may be regarded as owing to electrical movements, the production of animal heat can only be suitably explained in the same manner; for it is known to electricians that the conducting wire acquires considerable heat during the action of the pile. M. de la Rive, the learned professor of chemistry at Geneva, was the first to seize the happy idea of referring the phenomena of animal heat to electric agency.

Dr. Edwards adduces some very ingenious observations in favor of the electrical theory of nervous actions; they are contained in a paper which was read before the Royal Academy of Sciences of Paris, in May, 1825, on "muscular contractions produced by bringing a solid body into contact with a nerve, without a galvanic circuit." The experiments consisted in passing a solid body along an exposed nerve, in the same manner in which we pass a magnet along a bar of steel which we wish to magnetise. In doing so, our object is not to act by pressure, or mechanical irritation, but rather to touch lightly various contiguous portions of the nerve successively; the sciatic nerve of a frog, which had been pithed, was exposed from ¼ to ½ of an inch, and a slip of oiled silk passed under it, to bring it better into view, and to render it more tense; the nerve was then gently touched with a slender rod of silver, and immediately the muscles were thrown into contractions; the same effects were produced when rods of copper, zinc, lead, iron, gold, tin, and platina, and even glass and horn, were used. It is proper, however, to state that iron and zinc rods caused far less vigorous movements than other metals; and that indeed the movements varied with the nature of the rod employed; but no satisfactory scale of the powers could be established. The question to determine is, whether the muscular contractions are attributable to electricity, (which we know is developed every time one body exerts a mechanical action on another,) or to some other agent, perhaps unknown hitherto to us? If it be electricity, we may presume that we shall be able to vary the energy of the contractions, according as the nerve is made a more or less perfect conductor;—in its natural condition, lying among muscles, which are excellent conductors, a great quantity of the electricity is lost and expended; but if we place under the nerve a non-conducting body, as oiled silk, the whole of the electricity will be concentrated in the nerve. This precaution is had recourse to also in galvanic experiments, when it is wished to excite muscular contractions by very small quantities of electricity, such, for instance, as are produced by the mere contact of two metals. The above fact is therefore a convincing proof that the insulation of a nerve renders it much more energetically susceptible; so that when a circuit is established by means of two different metals, very obvious movements of the muscles may be produced, where, had the nerve been left in situ, it would have been insensible to all stimuli. Now similar phenomena occur in the experiments in which the nerve is merely touched with a rod, as above detailed; and it was found that the effects were scarcely notable if the nerve had not been previously insulated. After having in vain attempted to excite contractions by touching the nerve while resting on muscle, Dr. Edwards found that they might still be induced if the oiled silk were placed beneath the exposed nerve; and he was able to cause them alternately to appear and to cease by employing at one time a non-conductor, and at other times a conductor below the nerve. It was observed that the contractions were most readily induced by a quick and light touch of the nerve with the rod. On the whole, Dr. E. concludes that the contractions in the preceding experiments were dependent on electricity.—*Med. Chir. Rev. from Dr. Edwards on Life.*

3. *Treatment of Inflammation of the Lungs, by Large Doses of Tartarized Antimony.*—The following is a resumé of the experience of Dr. Munaret on this subject, taken from the Gazette Médicale, wherein the details are published.

*Number* of cases of acute inflammation of the respiratory organs, treated between the 28th of July, 1831, and the 15th of January, 1833, thirty-seven—viz. pleurisies and pleuro-pneumonies, 22; pneumonies, 15—which is about the rate of one case for every fourteen days.

*Seasons.*—Spring, 6 cases; summer, 8; autumn, 3; winter, 20.

*Sexes.*—Women, 17; men, 20.

*Ages.*—Among the females, between ten and twenty, 2; between twenty and thirty, 6; between thirty and forty, 4; between forty and fifty, 2; between fifty and sixty, 1; between sixty and seventy, 2.

Among the males, between ten and twenty, 6; between twenty and thirty, 3; between thirty and forty, 4; between forty and fifty, 6; between sixty and seventy, 1.

*Results.*—Recovered, 34; died, 3—viz. a blind idiotic girl and paralytic woman, affected for a long time with organic disease of the lungs; a woman who was doing well, when some other medicine was substituted for the tartar emetic, unknown to Dr. Munaret.

*Description of the Method.*—In most patients who are of sanguineous temperament, the practice commenced with a bleeding at the arm, repeated according to circumstances. In the more aged and feeble, the application of leeches to the chest was preferred. The *Rasorien* potion was administered thus:—

No. 1.—Distilled Water, ℥v.; Tartarized Antimony, gr. v.; Laudanum, gtt. v.

No. 2.—Distilled Water, ℥v.; Tartarized Antimony, gr. viii.; Laudanum, gtt. viii.

No. 3.—Distilled Water, ℥v.; Tartarized Antimony, gr. xii.; Laudanum, gtt. xvi.

A tablespoonful every two or three hours; cold water in abundance during the intervals.

As the disease declines, blisters, squills, &c.

*Progress of the Disease.*—Eleven days the mean duration. Diaphoresis is the constant indication of the medicine acting favorably; vomiting alone, or accompanied by purging, fourteen times in thirty-seven—viz. in eleven women and three men. A few drops of laudanum added to the potion, overcomes this effect. At other times, and indeed more frequently, purging takes place without vomiting, and without aggravating the principal affection.

*Doses of Antimony.*—From five to sixty grains, and upwards, in three days; mean quantity during the treatment, sixteen to twenty days.

*Precautions.*—Patient and those about him to be made acquainted with the probable effect of the medicine, otherwise it is apt to be discontinued in the absence of the practitioner.

*Inference.*—Tartar emetic, administered in large doses, and judiciously continued, with antiphlogistics and derivatives, is, to acute inflammations of the chest which are not complicated, what quina is to ague.—*Boston Journal.*

4. *Gonorrhœa and its Treatment.*—It is said to be a very easy thing to cure a gonorrhœa. We have not found it so. One physician will tell you that such a mixture always cures it in five days; another has a preparation that will do the work in three; and a third is so unfortunate that he can seldom entirely remove the complaint in less than a week. Authors of books allow us a month; writers in the periodicals sometimes confess half that time; but the proposer of a new remedy is seldom satisfied with anything more than a day or two. For ourselves, we have treated cases of all kinds, and at all stages of the disease. We have tried all the remedies that have been proposed for the last thirteen years; and when an oil or an extract, a pill or a draught, a simple injection or a patent wash, has been gravely pronounced, on good authority, to have removed the disease in a week, we confess that the remedy has been seized with a great and perhaps foolish degree of confidence, and tried to our heart's content—content, not with its efficacy, but its total inefficiency. Treatment physiological, and treatment empirical, have always failed to cure one full half of all the cases that we have had the misfortune to encounter. The discharge will often disappear in a day or two, or a week or two, and it is in this state of cases that they have probably been published; but it almost invariably comes on again, and, as we before remarked, full one half of our cases have been protracted for five or six weeks, or terminated in comfirmed gleet.

There is one remedy yet to be tried here, which, like its predecessors, has recently been found, abroad, to surpass all others in the rapidity and effect with which it accomplishes its work. It is an injection of the nitrate of silver, in solution. This practice, the reader will recollect, is condemned by Carmichael, as extremely harzardous, and endangering the sanity if not the integrity of the bladder and its appendages. But it is well known that the precise nature of the action of this remedy on the mucous membrane, has not been understood until very recently. Twenty years ago, a proposition to rub inflamed tonsils or fauces every two or three days with a stick of lunar caustic would have been deemed sufficient evidence of unwarrantable rashness in a physician, if not absolute insanity or ignorance. Now the practice is most common, and its perfect safety and curative effects every medical practitioner has learnt from his own experience. It is neither surprising, then, that Carmichael should have condemned this proposal eight or ten years ago, nor that it should be now renewed and carried into practice—with what result, we have too much reason to doubt until it has undergone the test of trial under our own eye. Mr. Lucas, an Irish Surgeon, who has recently thus treated this disease, appears to have had a very just view of its obstinate resistance to ordinary remedies; and he says, with regard to these injections, 'the results of my experience as to its efficacy convinced me, that if it be used with a cautious hand, and at a proper time, no bad effects will result, and that this hitherto almost intractable disease will be brought still more under the control of surgical skill.' Those who have used the nitrate of silver most extensively, as a local alterative, are most established in their conviction of its power in subduing diseased action, and the safety with which it may be usedon almost any portion of the mucous membrane within reach. Of all parts, that which is diseased in gonorrhœa is perhaps the one in which the greatest prudence will be required. But if the caution referred to by Mr. L. is uniformly observed, we should regard the remedy as perfectly safe, and as promising more than any other of recent origin.

Ten grains of nitrate of silver, dissolved in one ounce of rose water, constitute the injection. The urethra should be compressed with the left hand, about two and a half inches from the orifice, to prevent the solution from passing further down the passage, and then the solution injected freely over the diseased surface, by means of a small syringe of bone or ivory. In the cases related, this injection was made at 2

o'clock, and again ten hours after—and here the matter ended. We will offer the details of a single case, which was drawn up by a medical patient, and which appears to be a fair illustration of this treatment.

'Two days after connection, an unusual sensation at the orifice of the urethra directed my attention to the part, and I perceived a slight discharge. Twenty-four hours after, it increased, but supposing the person I had connection with would not have deceived me, I hesitated to use a remedy. The day following, the discharge was so great, of a yellowish color, accompanied with scalding, as to place beyond all doubt the existence of the disease. The nitrate of silver injection was used at two o'clock P. M., and at twelve that night. The following morning the discharge ceased, but there was no appearance of the purulent drop you led me to anticipate. At the end of two days, when the irritation caused by the injection had ceased, the gonorrhœal discharge returned. The injection was used again for two turns, as before, and at the same hours. The morning following, on pressing the urethra, I forced out a thick drop of purulent matter; no further discharge followed, and the pain gradually subsided.'

In gleet, this injection totally failed.—*Boston Journal.*

5. *The Anodyne Metallic or Galvanic Brush, (Scopula Anodyna Metallica. Annales Scholæ Clinicæ Medicæ Ticinesis. Auctore Francisco Nob. Ab. Hildenbrand, M. D. Paviæ,* 1830.)—Under this name, Francis Ernest Von Hildenbrand, Professor of Pathology and Practice of Physic at Pavia, describes a remedy rather singular, for the cure of various neuralgic affections. It consists simply of a bundle of metallic wires (*fascis e filis metallicis confectum,*) not thicker than common knitting wires, firmly tied together by wire of the same material, so as to form a cylinder about four or five inches long, and one inch or three-fourths of an inch in diameter. This is applied to the pained part, previously moistened with sea-salt, when it produces relief so instantaneous, it is said, that it appears to the patients like the effect of a charm. Occasionally the pain is immediately entirely extinguished, with the accompanying effect of a peculiar sense of emanation from the spot to which the brush is applied, causing the patients to believe that the pain is truly extracted by this method. On withdrawing the brush, the uneasiness occasionally returns, but in a more endurable form. The longer the application is continued, the more decided is the effect obtained; and phenomena so singular have resulted from its application, as even to astonish intelligent persons quite on their guard against any magical illusion.

In illustration of the remedial effects of this agent, Heldenbrand mentions the following case, which he designates as altogether singular and wonderfuld. A man of thirty, a porter by occupation, afflicted with violent periodical tic douloureux of the face (*metopodina,*) was admitted into the clinical wards of Parva. On applying the metallic brush over the left frontal nerve, the pain immediately disappeared from that one, but fixed on the corresponding nerve of the right side, which had been previously free from pain. The very moment at which the brush was removed from the left frontal nerve, the pain returned to its original seat, and there remained, though already remarkably abated in intensity. By applying a metallic brush to each supra-orbital nerve simultaneously, the Professor banished the original nerve-ache of the left side, and at the same time prevented it from appearing in the opposite one. The same moment, however, a humming noise arose in each ear, and this also immediately ceased on the brushes being removed, when the nerve-ache returned immediately, though in a very mitigated form.

In order to obtain the desired effect from the use of the anodyne brushes, Professor Von Hildenbrand impresses the necessity of determining, as accurately as possible, the nature of the *neuralgia*, or the pathological state of the affected nerve. If the pain is merely *nervous*, that is, proceeding from subversion of the equilibrium between the *dynamic factors of the sensitive life*, as the Professor, in imitation of his father, expresses it, without material changes having taken place in the affected part—in which case it attacks periodically, like an intermittent disease, and leaves intermissions entirely void of pain—then the efficacy of the metallic brush may be pronounced to be almost infallible. But if, from the pain being uninterrupted, or at least void of perfect intermissions—from its aggravation under pressure of the part, from the conjunction of redness, heat, or swelling—there is reason to believe that the proximate cause of any case of facial neuralgia or hemicrania, consists in a state of active congestion, or sub-inflammatory irritation—then the metallic brush affords no benefit, nay, sometimes may augment the intensity of the pain. By these means Professor Hildenbrand thinks that the metallic brush, while it maintains at least a palliative therapeutic property in neuralgia of spasmodic character, may, in doubtful cases, furnish an auxiliary diagnostic sign, by the aid of which sub-inflammatory congestion may be distinguished from simple nervous erethism.

In the first experiments performed by Professor Hildenbrand, he employed brushes which where intentionally constructed of two kinds of metal; for instance, silver and copper wire, copper wire and zinc wire, or zinc wire and brass wire, the individual wires being mutually mingled and blended, on the supposition that electricity or galvanism, evolved by the contact of heterogeneous metals, might be the beneficial and sanative agent. He afterwards ascertained, however, that bundles of wires of one and the same metal produced an effect scarcely less speedy, but lost their anodyne influence as soon as they were covered by rust or verdigris. He further ascertained, that solid metallic bodies produce analogous effects, but in a much feebler degree than the numerous acuminated points of the bundle consisting of metallic wires. The nature of the metal, he adds, seems to cause no difference; for brushes of iron wire produce the anticipated alleviation in as great a degree as those of copper wire. If he could trust his observations, however, he thinks that he perceived a greater degree of anodyne virtue in copper, iron, and gold, than in other metals.

Admitting that the effect is constant, to explain the theory of its production Professor Hildenbrand does not hesitate to deduce it from the laws of electricity. The original nature of metallic bodies, which are remarkably good conductors of electricity; the rapid action of the brush, if the aching spot has been previously moistened by the saline solution; the remarkable tendency of pointed bodies in attracting electricity; and the sense of emanation, and an agreeable coolness, combined with manifest alleviation of pain admitted by the patients, he regards as no trifling arguments to infer, in the disordered and aching nerves, a certain degree of *electric plethora*, or accumulation of animal electricity, which my be discharged by the application of a suitable conductor. This hypothesis, he lastly remarks, would accurately correspond with the notions delivered in his elements on the accumulation of the imponderable Biotic principle in various parts of the nervous system, as the proximate cause of nervous disorders which attack in paroxysms, and are dissipated by what he denominates autocratic explosions.—*Boston Journal.*

6. *Origin of Acephalocysts.*—The following case shows that a mechanical lesion may give to the part injured a disposition to form these parasitical productions. A girl, aged 16, of good constitution, though rather delicate, and who had not menstruated, fell while carrying a pail of water on her head, and struck the front of the thigh with so much violence that she could not rise for some moments. The part was fomented, and got so quickly well that in three days she was able to go about her usual avocations. A little swelling remained, but attracted no particular notice. This occurred in the summer of 1823. However, in June 1824 the swelling had become as large as a hen's egg, and continued to increase until, after severe exercise in the fields continued during the day, the tumor became so large and painful that the patient could neither walk nor stand. Dr. Held, of Fransbourg, by whom the case is related, was then consulted. He found an elastic tumor on the anterior part of the right thigh, and following the course of the rectus muscle. The thigh was about double the size of the other, but the skin retained its natural color. The surgeon at first took it for a lymphatic abscess, and recommended various remedies to relieve pain and inflammation. Nevertheless, fluctuation could not be perceived; but in its place a kind of elastic trembling, like that of firm jelly. However, caustic was applied, which penetrated to the fibrous covering of the thigh, but without reaching the tumor. The ulcer thus formed was kept open till December, but still the tumefaction increased, when at length, in February 1825, the tumor burst spontaneously. Pus, mixed with blood, flowed at first; and then, during five days, a yellowish serous fluid, with thousands of hydatids of different sizes, from a millet-seed to a hen's egg. The hydatids were spheroidal, colorless, neither adherent to each other nor to the adjacent parts. In nine months the swelling was quite reduced, and the limb entirely restored.—*Boston Journal, from Hecker's Litterarische Annalen.*

7. *Internal Use of Chlorine in Nervous Fever.*—Dr. Clemens, of Frankfort, almost always commences the treatment of typhoid affections by an emetic, to which succeed gentle purgatives (neutral salts) continued for several days; five or six evacuations being produced daily. The head is generally relieved by this; but if not, from twelve to twenty leeches are then applied to the forehead, temples, or behind the ears, with cold applications to the head, and a blister to the back of the neck. If, towards the fifth day, nervous symptoms set in, he prescribes two drachms of chlorine water in three ounces of distilled water, this mixture being taken a spoonful in the course of the day. In administering this medicine, it is necessary to avoid adding any kind of syrup, because it favors decomposition, and it is also necessary to keep the bottle covered with dark paper, and in a dark place. On the sixth day, Dr. Clemens has the patient somewhat more warmly covered, and discontinues the cold applications. During the six or seven days which follow, he makes little change in the treatment, except that the dose of chlorine is gradually increased to four or six drachms daily, in three or four ounces of distilled water. Perspiration generally continues from the sixth or eighth day, and two or three stools are procured. After the fifteenth day the chlorine is changed for a light infusion of valerian, and veal or chicken soup. At the end of three weeks decoction of bark is administered, and meat allowed.—*Boston Journal, from Medizinisches Conversations. Number XV.*

8. *Endermic Therapeutics.*—The method followed in France is as follows:—Apply to the skin, which we wish to deprive of its epidermis, a portion of ammoniacal pommade, which is made of equal parts of lard and the strong liquor ammoniæ, and renew the application in five minutes; in five minutes more the blister will have risen, and we then remove the epidermis, and sprinkle the raw surface with the medicament. Half a grain of acetate of morphia, applied on a blistered surface near the origin of the sciatic nerve, has, in 24 hours cured a severe neuralgia of the limb; and sulphate of quinine has, when similarly used, quickly put a stop to an ague, when the medicine could not be administered inwardly.—*Ann. de la Med. Physiol.*

9. *Mode of dressing a Stump after Amputation of the Thigh.*—While the assistant draws down with both his hands, the skin and muscles over the face of the stump, let the surgeon pass a bandage round the pelvis, and roll it regularly and firmly round the limb from the hip to the wound; apply the two cut surfaces neatly together, and retain them there by cross pieces of bandage, secured by a turn or two of the roller: over these put a piece of simple dressing and a pad of soft lint, which are also kept in their place in the same manner, without the use of any adhesive plaster, which are condemned as highly irritating to the wound.—*Journ. Complement.*

We quite approve of the above method, and have repeatedly observed the pernicious effects of applying adhesive plaster to large wounds. It gives us pleasure at the same time, to find that union by the first intention is now admitted into French surgery.—*Medico-Chirurg. Rev.*

10. *On the Changes which the Points of the Fingers undergo in Phthisis, &c.*—Hippocrates remarked that, in those who died of pulmonary consumption, the nails became bent—"phthisitis ungues adunci;" the assertion used to be called in question by many medical authors, but M. Pigeaux, who has directed his attention to this subject, and written a memoir on the etiology, symptomatology, and the mechanism of the fusiform development of the extremities of the fingers," fully confirms the truth of the aphorism. He examined the hands of 200 phthisical patients, and found that 167 of these were provided with "griffes Hippocratiques." Every tubercular patient does not certainly present this phenomenon, but in other diseases of atrophy the proportion is much smaller, not exceeding one in ten; it appears therefore, that a certain relation may be traced between thoracic maladies and the curving of the nails, although it occurs in other diseases, but certainly not so frequently. In 183 cases of diseases, not tuberculous, which had produced great emaciation, 17 exhibited the phenomenon of the curving of the nails in a very remarkable degree; of these 17, 9 were cases of organic affection of the heart—four of emphysema—two of asthma and catarrh, and two doubtful. An obvious dyspnœa existed in 13 of these cases, and also in almost every one of the 167 tubercular cases. I have no doubt that some connexion may be traced between all such maladies as create an impediment to the respiration or circulation, and the appearance of the nails alluded to, or, at least, between the former and the fusiform swelling of the last digital phalanx, with which the curving is generally associated. In 20 of the 167 tubercular cases, the patients had not lost their embonpoint. After many examinations into the cause of these phenomena, I am satisfied that the change in the points of the fingers precedes, and is the cause of, the curvature of the nails. Now this change consists chiefly in an œdematous infiltration of the pulp of these, by which the nail becomes mechanically forced out and forwards, and thus its end is curved round. As a general rule, it may be stated that the fusiform development of the last phalanx of the fingers, with the curvature of the nails, is generally indicative of the presence of tubercles, or of any derangement of sanguification.—If we notice particularly the change of form, we find that the swelling begins at the articulation of the 3d with the 2d phalanx—that it increases somewhat towards the root of the nail, which becomes the most projecting part, and then it tapers off to the end of the fingers the thumb and fore-finger are generally affected first. The progress of this affection does not depend so much on the "phases" of tubercular disease, or of organic affections of the heart, as on the influence which these have on the general state of "hematosis" and of respiration. I have observed it to increase, diminish, and even to vanish, with the removal of the cause which had produced it. It is more common in women than in men; it is much more rarely seen in the toe-nails, with the exception of that of the great toe, the swelling of which, and the consequent "growing of whose nail into the quick, often gives rise to much pain and annoyance." To impress his readers with the importance of the above appearances, as symptoms, the author says that he has, by attention to this particular, repeatedly been enabled to foretell the severity and danger of a pulmonary catarrh, of a pneumonia, &c. which were supposed to be of an innocent nature! he, therefore regards it as a very unfavorable sign; it exists, he says, in six-tenths of consumptive patients; and, on the whole, it is more frequently seen in those who still retain their embonpoint, than in those who are much emaciated. If the above remarks be confirmed by experience, it must be considered as a valuable adjunct in guiding our diagnosis. The anatomy of this change will be readily understood from what has been stated above; the nail, separated from the finger, appears very little, or perhaps not at all curved; but when in situ, it is found to be elevated and pushed forwards by the infiltrated pulp *underneath;* the bone is not altered.—*Archives Generales.*

We do not vouch for the entire correctness of the preceding details, but deem them well worthy of attention by all enlightened physicians. —*Medico-Chirurg. Rev.*

11. *Ingenious Method of applying Nitras Argenti to Ulcers of the Cornea, &c. &c.*—Take a silver female sound, or large silver probe, and heat an inch of its extremity in the flame of a candle; then rub lightly upon it a stick of the lunar caustic; the salt is immediately melted, and unites with the metallic surface, coating it with a thin layer of caustic; if it be too thin, we have only to repeat the same process. When the instrument cools, it must be wiped clean, and then it is ready for use.—*Bullet. de Therap.*

12. *Roseolous Eruption, Caused by the Use of Copaiba and Cubebs.*—A young man, who had formerly had syphilis, for which he had never been regularly treated, was received into la Pitie, under M. VELPEAU, for the treatment of an abscess of the abdomen, and a recent gonorrhœa. He was ordered a mixture of copaiba, cubebs, and magnesia; and after having taken it for six days, he began to experience a troublesome itching and burning over the whole head and front of the neck. The next morning there was a full eruption of roseolous patches over his body. These patches were scarcely at all elevated, of various sizes, irregular, some confluent, others distinct, and of a bright red color; they very much resembled the rash of measles. By discontinuing the medicine, and keeping the body cool, the eruption began to disappear on the third or fourth day. Three weeks subsequently, the patient having taken another dose of the same medicine, the eruption reappeared.—*Am. Jour. from Archives Generales, Nov.* 1831.

13. *New Theory of the Sounds of the Heart.*—Dr. Bouanet, in an ingenious thesis, explains the phenomena of the two sounds in the following manner. The first, or dull sound, is produced by the shock, or impulse of the tricuspid and mitral valves against the auriculo-ventricular orifices, and this shock is caused by the pressure of the blood on the valves during the contraction of the ventricles. The second or clear sound arises from the succussion of the blood in the distended aorta and pulmonary artery, backwards upon the semilunar valves, during the dilatation of the ventricles; the author considering that at this moment, there is a tendency to a vacuum in the ventricle, and that therefore there must be a strong impetus, or rushing back of the blood upon the closed semilunar valves.

The first sound is heard at the commencement of the ventricular contraction; hence the idea that the sound was owing to the contraction of these cavities; and as the sound is momentary, it has been believed that the systole of the ventricles is also of the same brief duration. The cause of its being duller than the second sound is, that the tricuspid and mitral valves are larger, and that the parietes of the auriculo-ventricular apertures are thicker than the semilunar valves and the coats of the aorta and pulmonary artery.

The pulse is not quite synchronous with the first sound, but follows immediately after it. To explain the cause of this, we must remember that the tricuspid and mitral valves close at the very commencement of the ventricular systole, whereas the blood is not entirely expelled till the contraction is over. Such, however, is the rapidity of the contraction, that the second sound is heard almost immediately after the first. The second sound is more clear and sharp than the other, because the valves, by the shock of which it is produced, are smaller and thinner, and because the parietes which transmit the sound are more sonorous than in the former case.—*Am. Jour. from Med. Chirurg. Rev. from Journ. Hebdom. No.* 97.

14. *Ergot.*—Dr. Boettcher, at Mendelurtz, in the duchy of Altenbourgh, believing that the different energy of various specimens of ergot might depend on the circumstance of its being collected before or after the cutting of the parent crop, obtained a quantity gathered before, and after the harvest. He sent the separate products to the minister of public instruction at Berlin, who remitted them to Dr. Kluge for clinical trial at the Maternite of that city. The substance was employed on fifteen females, all well formed, and with natural presentations. The following are his comparative results:—1st. The action of the ergot of rye collected before harvest is very energetic, while that collected after harvest is totally powerless. 2d. In many cases the remedy renders the forceps unnecessary, especially when the insufficiency of uterine force depends either on real atony, or on a spasmodic contraction of the neck of the uterus. 3d. The ergot gathered before harvest arrests uterine hæmorrhage. 4th. The dose is from thirty to sixty grains, administered in portions of ten grains every ten minutes.—*Am. Jour. from London Lancet, from the Allegemeine Medicin. Zeitung. Nov.* 10, 1832.

15. *On Ergot.*—An admirable essay on the use of ergot of rye in menorrhagia and metrorrhagia, has just been published in the *Bulletin General de Therapeutique*, by MM. Trousseau and Maisonneuve. We subjoin the conclusions they deduce from their observations, together with a tabular epitome of the cases from which they reason. These researches deserve the deep attention of all practical men.

"*Conclusions.*—From the preceding facts we deem ourselves entitled to conclude—

"1. That the ergot of rye exercises on the uterus a powerful, but transitory action.

"2. That this action chiefly concerns the fibres of the organ, and determines their contractions.

"3. That these contractions, constantly accompanied by pains, put a rapid stop to the menorrhagic discharges, on whatever cause they depend.

"4. That the state of the uterus in no respect influences theproduction of the pains.

"5. That the pains are observed even when a part of the neck of the uterus is affected with cancer.

"6. That the ergot of rye acts on the centre of the nervous system as a narcotic.

"7. That the resulting phenomena are slow but durable.

"8. That they are never serious or dangerous when we confine ourselves to combat the menorrhagia.

"9. That the dose may, without danger or inconvenience, be carried to several drachms in the course of four or five days.

"10. That in the treatment of menorrhagia, divided doses, given at equal intervals are to be preferred.

"*Lastly.* That we need be under no apprehension of commencing with a drachm dose, divided during the first twenty-four hours.

*Tabular Epitome of the Principal Circumstances of the Facts contained in the Body of the Memoir.*

| Cases. | Age. | Number of Children or, Miscarriages. | Duration of the Disease. | Cure in | Quantity of Ergot taken |
|---|---|---|---|---|---|
| 1. Menorrhagia | 18 | — | 13 days | 60 hours | 216 gr. |
| 2. Ditto. | 23 | — | 6 weeks | 7 ditto | 108 |
| 3. Ditto. | 30 | — | 15 days | 44 ditto | 168 |
| 4. Ditto. | 39 | — | 1 month | ¼ hour | 108* |
| 5. Ditto. | 41 | — | ditto | 6 hours | 204 |
| 6. Ditto. | 28 | 1 | 9 days | 18 ditto | 192 |
| 7. Ditto. | 23 | 2 | 1 month | 3 days | 240 |
| 8. Ditto. | 32 | 3 | 9 days | 4 ditto | 132 |
| 9. Metrorrhagia | 36 | 2 | 8 days | 24 hours | 180 |
| 10. Ditto. | 30 | 5 | 6 hours | ½ hour | 51 |
| 11. Ditto. | 30 | Several | 7 days | 10 days | 192 |
| 12. Ditto. | 35 | 10 | 4 days | 5 days | 288 |
| 13. Carc. uteri | 49 | — | 36 hours | 36 hours | 120 |

*Am. Jour. from Lancet, March* 30, 1833.

16. *Chemical Analysis of Ergot.*—In 103 parts of Ergot, Mr. Wiggers, of Berlin, has found—

| | |
|---|---|
| White oil matter - - - - - - - - | 35.0006 |
| Solid fatty matter, crystallizable, and of peculiar nature | 1.0456 |
| Cerine - - - - - - - - - - | 0.7578 |
| Fungous matter - - - - - - - | 46.0862 |
| Ergotine - - - - - - - - - | 1.2466 |
| Vegetable ozmazome - - - - - - - | 7.7645 |
| Sugar - - - - - - - - - - | 1.5530 |
| Gummy extract, with red coloring principle - - | 2.3250 |
| Vegetable albumen - - - - - - - - | 1.4800 |
| Acid phosphate of potash - - - - - - | 4.4221 |
| Phosphate of lime, and traces of iron - - - | 0.2822 |
| Silica - - - - - - - - - - | 0.1394 |
| | 102.0930 |

There are some remarkable points in the preceding analysis. In the first place, the presence of vegetable ozmazome identifies the ergot with the class of mushrooms in which this substance forms a considerable proportion. In this ozmazome seems to reside the power which promotes parturition. The ergotine is insoluble in water, and seems, from the experiments of M. Wiggers, to be the principle in which the poisonous qualities of the ergot resides. On several animals it has operated as a powerful irritant poison, while the ozmazome

*" In this case the discharge ceased in a quarter of an hour after the administration of the first dose 48 grains; the administration of the ergot was, however, continued until the third day."

produced no such effect.—*Am. Jour. from Lancet, from Alleg. Med. Zeit.* 10 *Nov.* 1832.

17. *Anthelmintic Emulsion.*—"Turpentine is now regarded as the most certain of our medicines for the expulsion of intestinal worms: it has been exhibited under a great diversity of forms, with a view chiefly to the modification of its nauseousness, or the increase of its specific powers: as combined in the subjoined prescription, it acts with remarkable efficiency:—℞. Infusi Sennæ, f. ℥x.; Syrupi Rhamni, f. ℥j.; Confectionis Scammoniæ, ℈ij.; Copaibæ, ♏xxx.; Olei Terebinthinæ Rectificati, f. ℥vi.—Ritè misceantur ut fiat emulsio, quæ horâ matutinâ sumatur.

"This the dose for an *adult*, having no contra-indicative symptoms: the patient should take it in bed, about four or five o'clock in the morning, and afterwards endeavor to sleep. Generally, within four hours, the medicine begins to determine cathartic effects: if these do not then commence, a small tea-spoonful of the confection of scammony, or half an ounce of castor oil, will induce alvine evacuations; after each of these, some drops of an essential oil, or of the aromatic spirit of ammonia, or a little brandy in water, will counteract the tendency to squeamishness and exhaustion which the terebinthinate medicines usually produce. In cases where invermination is indicated by general symptoms, though worms have not been discovered in the patient's dejections, this medicine often accomplishes the dislodgment of these reptiles and a removal of the disorder which their presence had occasioned: in all other cases, the results of its administration are invariable and salutary. For the destruction of tæniæ, the emulsion should be exhibited at least four times, at moderate intervals; its repetition, within a fortnight, seldom fails of eradicating the other kinds of parasitic animals by which the alvine canal is infected. The use of this vermifuge, should be accompanied and followed by a course of medication with bitters, iron, and mild resinous aperients with aromatics."—*Am. Jour. from Med. Chir. Rev. July,* 1832.

18. *On Infantile Convulsions. By Professor Graves, of Dublin. Extract from a Clinical Lecture.*—When we consider the convulsive affections of the infantile period, we find that they may arise from a variety of causes. In the first place, they may be produced by the process of dentition. Some persons seem to think this impossible: but it is not only possible, but true; for teething is capable of exciting a very great degree of irritation in the system. We also observe that an irritable state of the brain, accompanied by a hydrocephalic tendency, will produce convulsions; but in very many instances, particularly in children of the ages mentioned above, they proceed from intestinal irritation. Of those forms which spring from the irritation of dentiti n, or of cerebral excitement, I do not intend to speak, as, on these matters, the standard medical works furnish abundant information. I shall restrict myself, therefore, to some observations on those convvlsions which depend on intestinal irritation. As such convulsions frequently arise from causes which affect digestion, and produce a change in the mode of nutrition, they appear very soon after birth. The animal, which but a short time before was nourished by the placenta, is now supported by ingesta; and hence, from this sudden change, if there be any source of irritation existing in the system of the child, or in the nature of its food, an unhealthy state of bowels rapidly ensues. To the consequences of this affection, manifesting itself so soon after birth, nurses have given the name of *nine-day convulsions.* Again, when another change is made, and the nurse's milk is left off, children are also liable to convulsive fits, and these are the convulsions of ablactation. In fact, at any period during the first year, infants are very apt to get convulsions, from various causes. If the mother uses an improper kind of food or drink, or gets into a bad state of health, or be strongly affected by mental emotion, the quality of the milk will be suddenly changed.* Under all these circumstances, or if the child be over-fed, (a very common fault,) the bowels get out of order, the whole intestinal canal is thrown into a state of irritation, and convulsive fits succeed.

It is necessary to be more explicit on this subject. When you are called to treat a case of infantile convulsions, bear in mind that they very frequently arise, particularly during the first six months, from the cause before mentioned, and this should, therefore, claim, at once your attentive consideration. I remember the time when it was the common practice to treat every case of convulsions as if it were an hydrocephaltic attack, and when antiphlogistics, calomel, and cutaneous irritation, were the indiscriminate means employed in combating every form of this disease. If a child happened to get a convulsive fit, it was immediately said, here is inflammation or congestion of the brain; and leeches were applied in successive relays, calomel given in large doses, egg-shells, crabs' eyes, magnesia, and other absorbents administered, and the unfortunate infants cruelly tortured by the repeated application of blisters to the scalp. I have seen cases where this blistering was carried to such an extent, that the child had not a place to rest its head upon. It is to Dr. Gooch we owe the valuable discovery, that there is in children a state of heaviness of head and torpor, accompanied by a tendency to convulsions, in which depletion cannot be employed, and where narcotics and even stimulants may be used with advantage. Dr. Locock asserts, that convulsions of this nature may be recognised by the depressed state of the fontanelle, an assertion which I have not yet verified. With respect to leeching, I have to remark, that a single leech to an infant is equal to a bleeding in an adult; and yet how often have we seen children leeched and leeched, until, becoming pale and exsanguineous, they sink as much from loss of blood as from the effects of disease.

With respect to the causes and periods of indigestion in children,

* It has been lately proved, that the custom adopted by some, of keeping the child at the breast for a year or a year and a half, is both unnatural and injurious. Every child should be weaned when nine months old.

I have already spoken. There is one point more which I wish you to hold in memory. Milk is a compound fluid, a beautiful emulsion furnished by the hands of nature, in which sugar, oil, and curd, are blended with a certain proportion of water. Now, when a compound fluid, such as milk, enters the stomach, and is submitted to the process of digestion, those parts which are soluble in water are absorbed, and those which are not, become first coagulated, and afterwards undergo resolution in the gastric juice. Thus, while the water and sugar are absorbed, the curd of the milk is separated from it by coagulation, and forms a solid substance, which is acted on by the stomach, and becomes dissolved by the agency of the gastric juice, and in this way contributes to nutrition. Not a particle of the milk, however, ought to enter the duodenum until it has passed through the usual process of digestion. As the first step to the accomplishment of this is the coagulation of the curd, this occurrence takes place with extraordinary rapidity: and it is a sign of health if the milk be thrown up in this state immediately after it has been sucked. The rennets of young animals give striking evidence of this power. But if it should happen that the stomach does not act properly, and the curd remains undissolved, what is the consequence? The curd passes into the alimentary canal in a condition different from that in which nature intended it should, and consequently produces intestinal irritation. None of the purgatives given to children are attended by half so much griping as this substance. This explains the phenomena which, in such cases, present themselves to our observation. The child becomes griped, irritable, and feverish, his tongue is loaded and white, he gets restless, and now and then utters a shrill scream. In this way the disease may go on for a considerable time; as the child is dropping asleep, he starts suddenly and screams out, bends himself in the form of an arch, and throws his head back as in opisthotonos. I have seen children in this state for a week. The physician, or nurse, gives castor oil, or some other purgative, and a great quantity of the curds are passed, and surprise the child's relatives. On examining the discharge, you find it consisting of lumps of different sizes, colored imperfectly with bile, and having a burnt appearance; on breaking them up, you perceive them to be white internally, and consisting of indigested curd. You remove these by purgative medicine, and the child gets well. Now, we all can do this; it is clearly laid down in books: you are told to examine the egesta, and give purging medicine where it is necessary. But there is one fact which has not been noticed. When you have treated the child in this way, and the attack has been cured, if the child be very strong, when put to the breast again, he may go on well, and you have no futher trouble, but if he is weakly, or of an irritable habit, when he is brought back to the suck again, or spoon-fed with milk, the same process of imperfect digestion takes place, and he gets another fit. The physician is again called in, and repeats the purgative, and the child gets better a second time; and, in this way, the physician goes on giving medicine, and the mother giving milk, and every body wonders at seeing what a quantity of foul stuff passes from the bowels. How are you to avoid this? By making infant abstain from milk in any shape for twenty-four hours, sometimes for the space of two, or even three days. It is incredible how small a portion of milk, even in the most diluted state, will keep up this disease, acting like a species of poison on the intestinal mucous surface. You know, that animal poisons, such as variolous, or vaccine virus, will affect the system, even when applied in a state of extreme dilution, and you can therefore conceive, that a small portion of milk will operate in this manner. I attended a case of this disease some time ago; the child had a relapse, and, on being called in again, I asked the mother whether she had given it any milk, and she told me scarcely any. I am always suspicious where I hear the word *scarcely* used; and, on requesting to see the kind of food she had been administering, she handed me a bowl of barley water, with the usual proportion of milk and sugar in it: it is in this way that we see the disease prolonged week after week by the prejudices of the nurse and the ignorance of the physician. Well, if you forbid milk altogether, what will you give the child? Let him have chicken broth, barley water, thin panado, veal broth, or whey. How long are you to continue this? The number of days will depend on the power which the child possesses of regaining the proper tone of the stomach; some children will have the stomach out of order to-day and well to-morrow, and the length of time you are to keep up this diet will vary considerably. When you are called, therefore, to a case of convulsions, inquire into the history of its symptoms, the nature of the alvine evacuations, and the quality and quantity of your patient's food; and if you find that, before the attack, the child's bowels have been in a bad state, that they have been for some weeks inclined to be loose, or that the stools are, at the time, similar in color and consistence to what I have described, (though, by the by, you are often told that every thing is quite right when it is not the case,) you will then be able to judge properly of the nature of the case, and, by giving aperient medicines, you will probably not only cure the disease, but also prevent a return of the convulsions. Sometimes, however, the convulsive fits will remain after the irritating sordes have been removed by purgative medicines. Absorbents are next made trial of. These have a very beneficial influence in many cases, they can do no harm, and where acid is present, (and this occurs in the stomachs of children to a greater extent than in those of adults,) prove mildly purgative. But if the convulsions continue, what else will you prescribe? I remember attending, not long since, an infant, about three or four months old, who had been for some time under treatment for convulsions. Leeches had been applied to the epigastrium; it got calomel, castor oil, and hydrargyrum cum creta, absorbents, aperient and fœtid enemata, and blisters to the vertex and stomach. Still the convulsions went on. Well, what did I do? I prescribed the following mixture,—℞. Spirit. terebinth. ʒj.; olei ricini, ʒiv.; syrupi papaveris albi.; mucilaginis g. Arabici, aquæ fœniculi, āā. ʒij. Of this mixture, when well shaken, exactly ʒj. was to be given every third hour; and what was the result? It operated on the bowels, and produced a copious discharge of urine, a marked improvement took place, and towards evening the convulsions entirely ceased.

My friend, Dr. Brereton, has, in similar cases, after the bowels were evacuated, succeeded in preventing a recurrence of the convulsions by means of the following mixture, suited to a child six months old:—℞. Olei anisi, gt. iv.; Sacchari albi, gr. x.; intimé misceantur et adde Aquæ fontis, ℥ij.; Pulv. rhei, gr. x.; Carbonat. magnesiæ, ℈j.; Tincturæ opii, gt. iv.; Spirit. ammoniæ fœtid. gt. x. Sumat cochleare j. medium, tertia q. q. horâ.

It is to be observed, that much caution is necessary in giving such combinations containing opium to infants, but there is a period when depletion ceases to be useful, that a mixture like this will prove the most effectual means of curing convulsions. In such cases of convulsions, in addition to the use of purgative medicine, prescribing the mother's milk, and giving spirit of turpentine, you may, during the first twenty-four hours, while the child is strong, order a warm bath, applying, at the same time, a sponge dipped in cold water to the head; or, if the child be weak, incline its head over the side of the cradle, and use the cold sponge and you will find that it will diminish the fit.—*American Journal, from Lond. Med. and Surg. Journ. January* 26, 1833.

18. *On the Employment of the Chloride of Lime in the Treatment of Psora.*—Professor Fantonetti, of the University of Pavia, has lately published a statement of the happy results obtained by himself, both in private and public practice, from the application of the chloride of lime in the treatment of psora. The Professor has treated eight cases of itch, all of which were received into the Hospital of Pavia, about the same period with this remedy. Out of this number, five were cured in from six to eight days from the commencement of the treatment, and the rest in a few days more.

The manner of using it, is to prepare a lotion, composed in adult cases of from one ounce to an ounce and a half of the chloride, to a pint of common water, and in children, of one ounce of the chloride to the same quantity of water, with which the parts affected are to be washed three or four times a day. Every third day the patient should take a warm bath, for the double purpose of cleansing the surface of the body, and washing off the crust of carb. lime, which may adhere to it. The warm bath moreover, tends to sooth the irritation, which this remedy sometimes occasions, as when the quantity of the chloride has been too great in proportion to the water, or its application too frequently repeated, or when the skin itself was originally in a state of irritation.

Professor Fantonetti assures us, that in almost every case of psora treated in this way, a cure is effected in eight days time from the commencement of the treatment, and he recommends it to the profession as the most certain, the most prompt, and at the same time the most economical of all the remedies with which this disease may be treated. These results of Mr. F. should particularly attract the attention of the profession, since similar ones had previously been obtained in France by the exhibition of this article. M. Derheins, an apothecary, residing at Saint-Omer, published in 1827 a paper on this subject: and to him, therefore, is due any credit that may be attached to the introduction of this remedy into practice; upon the utility of which our professional brethren will have to decide. M. Derheins, as well as M. Fantonetti, assures us, that he has cured some of the most inveterate cases of the disease, with it alone, after they had resisted a host of other remedies. He employed a lotion consisting of three ounces of the chloride to a pint of water, with which the diseased parts were washed three or four times a day. The average period necessary to effect a cure in his hands, varied from six to ten days. He had however remarked, that the cure was more prompt when, instead of the common solution of the chloride in water, he employed a liquid chloride, obtained by passing a stream of chlorine gas through a mixture of lime in water, until the gas was in excess. We should, moreover remark, that the statements given above are supported by the following facts. In 1810, the Spanish prisoners who were crowded together in large numbers at Flessingue, were attacked by fevers of a very fatal character, which soon thinned the ranks of these unfortunate beings, the most of whom at the time labored under the itch. Water impregnated with chlorine was used in order to diminish the contagious tendency of the fevers, and M. Cluzel, the apothecary, observed that all those individuals affected with psora who washed their hands in the chlorated liquid, were very much benefited by it, and more than one attributed their cure of this disease to the employment of this simple remedy alone. However this may be, it is certainly an interesting fact, that in repeating the experiment of M. Derheins, which were published M. Chevallier in his treatise on the chlorides, that M. Fantonetti met with similar beneficial results. The observations which have been made upon this article, should induce the profession to set the point relative to its great efficacy at rest, by at once repeating the experiments of MM. Derheins and Fantonetti. If the cure be as prompt as they state it to be, the introduction of the remedy will certainly prove one of the most important improvements in the treatment of this disease; for, according to Melier, the average period required by every other method of treatment employed in psora to effect a cure, is twenty days. The odour of the chloride of lime is by no means so disagreeable as that of sulphur, (the remedy usually resorted to,) nor does it stain every thing with which it comes in contact, as the latter article, which is almost always combined with some fatty substance, does. If, therefore, it proves as successful in effecting a cure as the sulphur has, it certainly should be preferred to this article in practice. —*Am. Journ. from Bulletin de Thérapeutique.*

19. *Chronic Bronchitis.*—Professor GRAVES has been treating a case of this, in which he suspected dilatation of the bronchial tubes, with an emetic every day, after the plan recommended by Dr. Fothergill, and lately by Dr. Elliotson.—"In cases like this," says Professor G., "there is a great quantity of mucus in the bronchial tubes, the lung becomes loaded and respiration oppressed, and the best thing you can do is to give an emetic, which produces the most beneficial effect on respiration. That the emetic may not interfere with the man's digestion, I give it in the morning, about four or five o'clock, when he awakes. Such persons are generally awakened at an early hour by the cough, which is brought on by the quantity of mucus accumulated during sleep, and continue to cough for three or four hours with great distress. This is the time when an emetic is given with the best effects; it unloads the lungs, shortens the fit of coughing, and gives very speedy relief, at the same time that it does not interfere with the patient's meals, for the stomach will be quite well before breakfast."—*Am. Journal, from Lond. Med. and Surg. Journal. April*, 1833.

20. *Method of Applying Nitrate of Silver to Ulcers of the Cornea.*—A very ingenious method of preparing nitrate of silver for application to ulcers of the cornea, is described in the *Bulletin de Thérapeutique.* This consists in heating an inch of the extremity of a silver probe, and then lightly rubbing it on a stick of lunar caustic. The salt is immediately melted, and unites with the metallic surface, coating it with a thin layer of caustic; if too thin the process is to be repeated. When the instrument cools it must be wiped clean.—*Am. Journal.*

21. *Chloride of Soda in Burns, Scalds, and Black-Eyes.*—Mr. Holt recommended at a meeting of the Westminster Medical Society, the chloride of soda as the best application he is acquainted with in the cases just enumerated. Mr. H. stated that he was called to a child that had pulled a saucepan of boiling water over its face and chest, on Monday, by which the whole of the cuticle was destroyed. He had four ounces of the solution of chlorine and water made up and applied, and subsequently chlorine mixed with unguentum cetacei spread on the parts. The result was, that by the following Saturday, the injured part was perfectly well, while a small portion on the back, treated by other means, was unhealed for a fortnight. He particularly recommended the use of the chlorine where vesication had not yet taken place; and pledged his veracity, that if applied immediately after a scald had occurred, not the slightest scar would remain. Where the skin was broken, it was his custom to make the lotion of four ounces to a pint of water, and the ointment of a consistence not quite so thin as cream. Amongst other cases he cited was one of a solicitor, whose hands were much blistered in putting out the flames of his bed-curtains, fired by his servant. He (Mr. Holt) sent a couple of quarts of the lotion to this gentleman, had it poured into soup plates, wrapped his hands in lint, as no skin was broken, placed them in the plates, and kept them there some time, and the next morning his hands were so perfectly well, that only one small dried patch of burn existed; and he was not out of his office an hour in consequence of the injury. An hour and a half had elapsed before the application. For the first few minutes the remedy increases the pain, but after that it produces ease. He would also state, that he knew of nothing so efficacious in a "black-eye" as the application of a solution of chlorine. It would disperse the awkward effects of a blow in that direction, almost like a charm.*—*Am. Journ. from Lancet, April* 6, 1833.

22. *Prolapsus Ani. Extract from a Clinical Lecture by Baron Dupuytren.*—Prolapsus ani consists of an eversion of the inner membrane of the rectum, which, forming a kind of invagination, descends within itself, and at length projects beyond the sphincter to the extent of two, three, four, five, or even six inches. Usually the intestine comes out every time the patient goes to stool; in other cases it descends only when the patient has remained in the standing posture for a long period; while, in other cases, the gut may become everted at any time, which shows that the relaxation is carried to a great extent, and that prolapsus takes place without any effort on the part of the person affected. In general the gut is easily returned; but sometimes the sphincter ani produces such a degree of strangulation, that the projecting membrane becomes at first dark-red, then black, and at length falls into a state of gangrene. Should you happen to see such a case of strangulation as I have described, you are to endeavour to reduce the gut in the following manner:—The patient must lie on the abdomen, and the pelvus must be raised conveniently by pillows placed between the thighs; some pledgets of wet lint are then to be placed round the base of the tumour, and gentle pressure exercised in order to reduce its volume. After this a compress should be placed on the centre of the orifice of the intestine, which is to be returned by gentle pressure into the abdomen. When reduction by this means is altogether impossible, we may be compelled to have recourse to scarification of the gut, but as the employment of cutting instruments may be attended by ulceration or inflammation of the large intestines, they are to be avoided as much as possible. The same observation is applicable to the use of leeches, which may be followed by external or internal hæmorrhage, ulceration, &c.

"But, though the reduction be accomplished, we have still to combat the tendency to prolapsus, which depends on the weakened action of the sphincters, or perhaps on certain causes producing strong contraction of the muscular fibres, inverting the large intestines, such as chronic inflammation. In the latter case, our first care will be to combat the inflammatory action, which gives rise to the disease; but when the prolapsus depends on a want of action in the sphincter ani, the best method of cure is that which has for its object the diminution of the cutaneous and mucous parts which surround the anus. The practical question to consider is, how can we best assist the action of the sphincters? Cold baths alone are frequently sufficient for this purpose; but the remedy is tedious, expensive, and requires

*In some of the northern counties of England, the working men treat a black-eye very beneficially, by sucking with the mouth, for a short period, behind the bruised man's ear.

constant attention. A better mode consists in removing some of the folds of skin, which surround the margin of the anus, so as to diminish the extent of the soft parts which dilate when the patients goes to stool, and to determine adhesion between the skin and neighboring parts. When the patient is placed in a convenient posture, the operator, holding in his hand a common forceps, (with the points rather blunt, so as not to pinch the skin,) seizes successively several of the folds of skin which surround the margin of the anus in a radiated manner, and with a scissors curved upon its flatside, he excise several of these folds as far as the margin of the anus, or even two or three lines further; if the prolapsus be considerable, and of long standing, it will be necessary to extend the excision an inch within the anus the sphincter ani must not be touched, as the operation concerns only the external tissues."

M. Dupuytren has never seen any hæmorrhage or unpleasant cirmustance accompany this. The pain of the operation instantly occasions a strong contraction of the sphincter ani, and the inflammation extends for a slight way to the neighboring tissues. Usually there is no stool for the first few days after the operation. About the eighth day the inflammation begins to subside, but the excretion of fæcal matter continues to produce pain and violent contraction of the sphincter ani. M. Dupuytren has never seen the prolapsus reoccur after the operation.

At the conclusion of the lecture, the Baron proceeded to operate in the manner he had described, upon a young child of three years and six months old. The gut projected about three inches beyond the margin of the anus, and came down every time the boy went to stool. M Dupuytren having returned the projecting bowel, nipped off with the scissors four or five bridles of the skin round the margin of the anus. The little patient lay perfectly quiet, and seemed to suffer so little pain, that immediately on getting up he offered to the Baron an orange, which he had received to keep him quiet, and pronounced his "adieu" with an undisturbed voice.

4. The boy lively, and seeming to suffer little or no pain. A plug has been placed on the anus, and supported by a bandage. No stool since the operation.

5. The little patient seems to suffer some pain to-day when he moves, but is otherwise quite well: has passed two stools since the last visit. The gut has not descended, or even approached the margin of the anus.

7. No descent of the gut; goes to stool regularly, but seems to suffer some pain.

11. The uneasiness has gradually diminished; no accident of any kind has occurred, and the boy was dismissed to-day apparently perfectly cured.

29. To-day the boy was brought by his mother to be examined at the hospital. There had been no return of the prolapsus, or appearance of the internal membrane at the anus, although for the last few days the child had been affected by a diarrhœa, which compelled him to go frequently to stool, and occasioned much straining.—*Am. Journ. from Lancet, April* 27, 1833.

23. *On the use of Cold Water in Cholera. By Dr. Hardwicke Shute, of Gloucester.* (*In a letter to the Central Board of Health.*)—My attention was early directed to the fact, that no good, or rather as it appears to me most decided injury was done by the administration of brandy, (or alcohol in any of its multifarious forms,) and even by the use of the stimulant emetics, when they were retained, as frequently happened in the advanced stages of collapse. In endeavouring to account for the deaths which commonly occurred in the course of a few hours, I was forcibly struck with the marked analogy which exists, as far as the state of the pulse and diminished animal heat are concerned, between the collapse of cholera and the impaired vital energy which results from starvation or long-continued exposure to excessive cold. Now it is an established fact in therapeutics, that the administering of a stimulus, disproportioned in strength or extent to the impaired vital energy of the system, is, under such circumstances certain death; and that mortification of a frost-bitten extremity uniformly results from the hasty application of too great a degree of heat, or other stimulant, to the affected part. The treatment which I have adopted in the second and third stages of cholera is founded on the analogy already mentioned, and on the general principle that the necessity of diminishing or absolutely withdrawing every kind of stimulus is greater in proportion to the degree of collapse, or sinking of the vital powers.

The circumstance which particularly directed me to the remedy I employ, was the thirst, the excessive and inordinate desire on the part of the patient for water, for cold water more particularly, and I may almost say cold water exclusively; all other liquids being taken with reluctance, if not absolutely refused. I shall therefore state in general terms, that the free and unrestrained allowance of cold water, which, in the most marked cases of recovery, was taken to the extent of some gallons in a few hours, is the circumstance to which I wish particularly to direct your attention. If I add to this the abstraction of all kinds of stimulant remedies, both external and internal, even to the exclusion of friction, or the application of heat in any form, I have stated in general terms the whole plan of treatment, which, as I shall show, has succeeded in twelve out of fourteen, if not in twelve consecutive cases of the third stage of cholera. I am fully aware that the number of cases is too few to justify any thing like a general conclusion; but you will, I think, agree with me that they are not too few to justify a more extensive trial of the plan proposed. And it is with this view that I now address you; hoping that through your influence such trial will be made on a more extensive scale than is in the power of an individual.—I shall now mention some particulars which appear to me calculated to throw additional light on the plan of treatment I propose, it being understood that my observations apply to the *second* and *third,* particularly to the *third* stage of cholera

"when the pulse at the wrist has ceased, or become almost imperceptible."

The windows of the cholera hospital in Gloucester are large and numerous in proportion to the size of the apartments, and are open night and day. The doors open immediately into the garden, and are not suffered to be closed, so that the patients may be considered as living in the open air. I may add that the fires are purposely kept so low as to influence as little as possible the temperature of the room. The covering of the patients is confined to a light blanket and rug; and it seldom happens that some part of the body, particularly the breast and shoulders, is not constantly exposed. Under these circumstances, a pint of cold water is offered to the patient, and very frequently two-thirds of this is taken at a draught. In what I consider the most favorable cases, vomiting is almost immediately produced, and the patient in two or three minutes again calls for and eagerly drinks the same quantity with the same result. This is often continued for hours, until gallons of water have been taken, and the greatest proportion, (but I conceive not all,) rejected by vomiting. In other cases the patient is too insensible to ask for the water; and under these circumstances, it is offered every ten minutes or quarter of an hour, and most commonly drank with avidity. If gruel or tea be offered, the patient most frequently refuses it, and generally speaking no kind of nutriment is taken in any form until the period of convalescence. I consider it of great consequence that the vital powers should be restored as gradually as possible; and it is of importance to remark that the progress towards recovery has been in all cases remarkably gradual and uniform. In the first six or eight hours no amendment can be observed except some diminution in the purple hue of the extremities; in the next six or eight hours there is a manifest improvement in the countenance of the patient, and more disposition to sleep, but often no restoration of the pulse or increase of temperature. In some cases the pulse has not been perceptible for twenty-four or thirty-six hours. From this period the pulse, the animal heat, and the secretions are very gradually restored, and at the end of forty-eight hours, or the third day from the commencement of the treatmeut proposed, the patient is convalescent, and in all cases without consecutive fever. I mention these circumstances particularly, in order that the practitioner may not become impatient. He should, inmy opinion, be satisfied, and make not the least alteration in the plan proposed as long as the patient is merely not getting worse.

It would be inconsistent with the professed object of this memorial to offer any remarks upon the pathology of cholera, or upon the *modus operandi* of the treatment here recommended. But I cannot refrain from briefly suggesting, first, the importance of knowing;—that in the collapsed stage of cholera, cold may be extensively applied to the coats of the stomach without diminution, to use the most cautious term, of the vital energy. Secondly, that cold so applied has a manifest tendency to check the serous secretion, or perhaps, more correctly speaking, excretion or exudation of serum, which characterizes the disease. Thirdly, the acknowledged effect of vomiting in checking diarrhœa, equalizing the circulation, and emptying the vessels of the liver, and consequently the importance of not checking a natural action which has a conservative tendency. Fourthly, the probable effect of the liquid absorbed by the stomach in restoring the fluidity of the blood; and the presumption that the saline treatment, both as applied to the stomach and by its use in venous injection, may owe its effect as much, probably more, to the fluid itself, than to the ingredients it contains. Fifthly, the great importance of the arterial circulation being restored as gradually as possible, and the fact of convalescence taking place without the consecutive fever, which so often proves fatal in the *fourth* stage—the stage of reaction.

This method of treatment I have used with success in twelve of fourteen cases, and I may say in twelve consecutive cases. The first case in which it was tried and proved successful, occurred about six weeks ago, soon after the first appearance of the disease in this city. In the four first cases of the disease which came under my notice, internal and external stimulants were liberally administered. The mustard emetic and bleeding were also had recourse to, and every patient died in less than twelve, one in six hours. I determined therefore, (having understood from my professional brethren that the saline treatment had equally failed,) to take the first opportunity of watching the disease uninfluenced by remedies, with the hope that I might thus ascertain, if possible, the natural effects of the constitution, if any, to relieve the disease. In the cases already mentioned, I had observed that the stimulants were taken with great reluctance, and in one instance of extreme collapse, when the patient was apparently unconscious of every thing, I was much struck by an earnestly-expressed desire, that no fire should be made in the room, directions to that effect having been given in her hearing. In two of these cases the thirst was, I observed, excessive, and very distressing to the patient.

Soon after this, I was requested by a medical gentleman of this city to visit a female, fifteen years of age, whose father was at that time lying in the house dead of cholera. It was a marked case of collapse, with constant vomiting and purging of a fluid like rice-water. The pulse at that time, about one o'clock P. M. was scarcely perceptible. Calomel and opium had been previously given. I recommended a very liberal dose of opium, with various stimulant antispasmodics and olive-oil, and visited the patient again at seven o'clock that evening. The symptoms at that time were much aggravated—the vomiting and purging not at all relieved—the pulse imperceptible—expression of countenance cadaverous in the extreme. In despair, I recommended ten grains of musk to be given every hour, to the extent of two scruples; and left the patient with the impression, which was also that of the other medical gentleman in attendance who was with me, that she could not survive many hours. Having reflected upon the case and still feeling anxious that relief, if possible, should be given, I returned in an hour with some green tea, and desired some to be made very strong and given. Whilst this was in preparation I visited the patient, and to relieve the thirst, which was excessive, presented a pint of cold water, the greater part of which was eagerly swallowed, and in less than a minute the whole was apparently rejected by vomiting. At this time I may observe, the symptoms were the same as last described. In two or three minutes after she *asked* for the water, which was drank, and immediately rejected as before. Having witnessed the continued repetition of the process for half an hour (during which time the tea had been offered, and refused as soon as tasted,) without any increase of collapse, and with the satisfactory circumstance of a change in the voice, which had been highly characteristic of the disease, I left directions that cold water should be given to any extent which the patient might desire. I was informed the next morning that the patient had taken some gallons of water; had been constantly sick after each draught; but had not been sick, or asked for water the last hour. I also ascertained that the window, which opened immediately upon the bed, had not been closed the whole night. At this time there was no perceptible pulse or increase of temperature, but the countenance was, I thought, not quite so cadaverous in expression. The same plan was continued.—In the middle of the day there was still no manifest improvement, but in the evening the pulse was perceptible. The next morning the pulse was decidedly improved, and on the following day the patient was convalescent, without any appearance of fever. If I have been tediously minute in the history of this case, I trust that it will be attributed to the great interest I must naturally feel in a case which terminated so satisfactorily, and which was the foundation of my future practice.

Soon after this, I was told by another medical gentleman that he was in despair, having lost seven cases of cholera in succession, and had just visited another, who, he supposed would be dead before night; and that he had done nothing but ordered small doses of capsicum, with camphor mixture. I found this patient in a similar state to the last—recommended the unrestrained allowance of cold water—heard she was better the next day, and found her convalescent the following day, but with indications of fever. This circumstance, however, was accounted for by the fact that beer and cider, as well as cold water, having, through the prejudice of the attendant, been frequently given.

My experience, subsequent to the cases mentioned, has been confined to the Cholera Hospital in Gloucester, which was opened a fortnight ago, and placed under my superintendance, with the assistance of a resident surgeon. The first four cases sent to the hospital which was then under my care, were all dead next morning. Since that period, of the forty-eight cases admitted, twenty-one have been discharged cured; eight I consider convalescent: eight are under treatment; and eleven have died. Of the eleven deaths, two might fairly be attributed to previous treatment, or other circumstances; three were in progress towards recovery, and relapsed from improper exertions on the part of the patients; two which were under three years of age, died under symptoms of cerebral congestion, after the pulse had been restored; one was more than sixty years of age, another about fifty, and extremely emaciated from previous disease or starvation; in one, the case was too advanced to admit of the plan being tried; and in another the patient was a notorious drunkard, and died a few hours after admission, with a gin bottle concealed about her person. If due consideration be given to these circumstances, the deaths cannot, I think, throw any discredit on the practice. I may add, that in all the cases great allowance must be made for the effects of previous treatment, as being most commonly directly opposite to my views of the disease. My experience hitherto justifies, in my opinion, the following conclusions, viz:—That in all cases the progress towards death is retarded by the water treatment; that when the irritability of the stomach is excessive or unimpaired, reaction will be established in a very great proportion of the cases; and that when the natural powers of the constitution are not unusually weak from extremes of age or other causes, such reaction will be followed by an early convalescence.—Any suggestion from those who are much more conversant with the disease than myself, will be received with gratitude, and any question answered to the utmost of my ability.—*Am. Journ. from the Edin. Med. and Surg. Journ. October*, 1832.

24. *On the Use of Ice and Chloride of Soda in Cynanche Maligna. By Samuel Jackson, M. D. of Northumberland, Pennsylvania.*—[*Communicated in a letter to the Editor.*]—In the last number of your Journal, p. 261, you have published an extract of my letter concerning the use of ice in cynanche maligna. This letter was written in April, 1832, and since that time I have had ample opportunities of testing this remedy. In more than forty cases I have found it highly useful, and without it I am certain more than one-half would have been lost. Mrs. Magill of Danville, Baskins of Selinsgrove, and Lotz of New Berlin, have all used it with the happiest effect. So necessary has it become at the last named place, that Mr. Laschelles, a benevolent gentleman of that town, has refused to let his ice be used even in his own family, thinking proper to reserve it all for the sick.

The patient ought to hold it almost constantly in the mouth, as directed in my former letter, and swallow the solution; but if he be not old enough to rest on his guard against swallowing the undissolved lump, it may be enclosed in a gauze bag. If the fever be high he may swallow small portions of it finely powdered.

If the child be too young to manage ice, we inject the coldest solution of it into the throat very frequently; and in these cases pounded ice may be swallowed if there is much fever.

I have often thought of using it externally, but somehow the only favorable opportunities of trying this mothod have been suffered to escape unimproved.—"Occasio celeris, experimentum lubicrum, judicium difficile," says the reputed father of physic, and as the disease has passed over us, I refer the experiment to others.

If you or your friends should try the ice, let me assure you that much will depend upon the incessant use of it night and day, till the inflammation has evidently yielded. Till this time the patient can take very little rest unless it be a mild case that may not require this uninterrupted repression and sedation. But in bad cases we must not suffer the disease to gain upon us during the night what it has lost under the use of ice the preceding day.

Every case of cynanche tonsillaris, or simple inflammatory sore throat which has fallen under our care for the last twelve months, has been treated with ice after bleeding, and with great advantage. And in one case of most severe aphthous sore throat, which attended a low bilious fever, that of Dr. Robbins, of Sunbury, he assured me it was the pleasantest thing he had ever experienced, and I may fairly add—the most useful. But even the comfort which this remedy brings, is not to pass unnoticed. Mr. Greenough's daughter, eight years old, was delirious in the cynanche maligna for many days, and the ice could not be used. During all this time, four days and nights, she swallowed not one tea-spoonful of any thing whatever, but was incessantly employed during all this time, unless when she might dose for a few minutes in washing out her mouth with ice water, and to this must be attributed her safety.

Chloride of soda is another most noble remedy in this wayward disease. We have begun the use of it as soon as the cineritious specks appear, if at the same time there be general prostration; but if the gangrenous process is slow, and there is abundant vivacity in the system, it may be omitted altogether, and the ice continued. The sloughs will gradually and surprisingly disappear as the general inflammation of the fauces abates.

When the gangrene is considerable, and the inflammation is at the same time diffused, we use the ice and chloride alternately. But when the system is sinking, and we have reason to suppose that collapse is at hand, the ice must be omitted and the chloride used very strong.

With respect to the strength of our medicine, we are at some loss.—Such as we have received from Philadelphia, we have used with from one part water to ten, according to the age of the patient and the state of the disease; but we are by no means satisfied with our experience on this subject.

In order to purify the stomach we have given in the advanced stage of the disease, large doses of calcined charcoal, such as a drachm three or four times a day to a child ten years old.

We have bled freely in some cases, given calomel purges, poured abundance of cold water on the head when there was delirium, and sometimes have put the feet into warm water, while cold was applied to the head. We believe that many who are lost in this disease perish of inflammation of the brain; hence we suppose that cups to the back of the neck would prove highly beneficial. Leeches to the throat, we are aware, have been used, but they cannot be generally attended to in the country. All these remedies are in trial among my medical friends, and when they shall have made their report, I will give you a more full account of our methods and their success, for the use of your Journal. Meanwhile, I hope that you will try these medicines, if an opportunity should offer, for I can assure you, that I have not lost one single patient of all whom I have myself exclusively attended. I began the ice with my first patient, my own child, and I have used it in nearly all the rest. A robust boy, four years old, son of William Zeclif, died, to the surprise of his friends, and without medical aid; I was soon called to the rest of the family, four miles in the country, where I found the mother, her sister, and three children, the youngest three months old, all very ill of the disease. Here was certainly a mortal disease, as it had already destroyed one of the family, but all the others recovered under the use of ice and chloride of soda. Mrs. Schuyler, of this town, lost one of her children of the disease at Pennsborough, twenty-seven miles above us. Here neither ice nor the chloride was used; but when the mother returned to Northumberland, her other three children were severely attacked with the disease, and they all recovered under the use of ice, without any other application to the throat. Daniel Gossler of this town, lost two children in whose cases neither ice nor the chloride was used, though under the care of a skilful physician, and great numbers died in the country above us, while the new remedies were yet unknown.—*Am. Journal.*

25. *On the Efficacy of the Cold Dash in Nervous and Convulsive Diseases. By Charles A. Lee, M. D. of New York.*—In the last number of the American Journal of the Medical Sciences, there is mention made of the efficacy of the cold dash in convulsions of infants, particularly in cases of hydrocephalus. It is perhaps not generally known, that pouring cold water in a continued stream upon the head is one of the most effectual remedies in most cases of nervous and convulsive diseases. In hysteria, epilepsy, chorea, delirium tremens, and catalepsy, I have employed this remedy with the most decided success, as well as in convulsions of children from teething.

The following case of catalepsy I relate, not only because the disease is one of rare occurrence, but to illustrate the effects of cold when applied in the manner above-mentioned.

In January, 1831, I was called to visit a coloured girl, aged fourteen, large for her age, and who had previously enjoyed good health, with the exception of slight pains in the right hypochondrium, extending to the spine, occasioned as was believed by a burn in that region, which occurred when she was a child. Latterly she had suffered much inconvenience from it, and the day when I first saw her, she had complained more than usual. I found her in the following situation. She lay on the floor on her back in a perfectly insensible state, incapable of moving or being roused by pinching, calling aloud, shaking, or even by running pins into her flesh. She lay in the exact position in which she fell, and had not been seen to move in the slightest degree. The pulse was 100, of natural force, eyes closed, respiration easy, and the skin of usual temperature. I raised her arm to an angle of forty-five degrees with the body; it remained in that situation. I bent the forearm on the arm; it staid bent. The same occurred to her other limbs. They were easily flexed, but it required considerable force to straighten them again. The eyes were closed, and the eyelids when raised shut in a short time. How long her limbs would have remained in the position in which they were placed I know not, but they continued thus while I was in the house, which was perhaps an hour. I noticed occasionally slight spasms in the muscles, but not sufficient to cause motion. She lay in this state fourteen hours, and then came out of it perfectly unconscious of any thing that had transpired. In about a fortnight afterwards she had another similar attack, from which she recovered in six hours. In three or four weeks from this time, she had a different attack, being violently convulsed, so that it required several persons to hold her, and prevent her injuring herself. Incurvation and recurvation of the body would alternately ensue with surprising rapidity. She foamed at the mouth, seemed unconscious of what she was doing, and sensation and voluntary motion were suspended. She had six or seven of these paroxysms, which lasted about twenty minutes each time, in the intervals lying as in the former fits. On pressure over the dorsal vertebræ she started as if it gave her pain, and moved her legs as long as the pressure was continued. She lay in this state twenty-four hours. I now directed cupping over the tender portion of the spine, and made an issue with concentrated nitric acid; this was kept discharging for several weeks, during which time she had no attack. In about a week after it healed she was again seized with a similar fit to the last, and lay *five days* perfectly insensible, without swallowing any thing. When she awaked she was but little inclined to eat, and as she had had no evacuation, digestion appears to have been in a measure suspended. The measures employed to shorten the duration of the fit appeared to have but little effect except venesection, which evidently was beneficial in several instances.

About seven months since she became pregnant, and has had three returns of the disease in that time, from which she has immediately recovered by pouring cold water in a continued stream from a considerable height upon her head. In no instance has it been longer than five minutes before she was perfectly restored. I have no doubt were it employed at the proper period it would prevent an attack; but as there is generally no warning, or *premonitory symptom*, this is impossible.—*Am. Journ.—New York, May* 18, 1833.

26. *On the Formation of Callus, and the mode of Remedying it when diseased or deformed. By Baron Dupuytren.*—There is perhaps no subject in pathological anatomy which has more exercised the sagacity of observers and the imagination of those who raise up hypotheses without experience, than the theory of the formation of callus. Two opinions have chiefly prevailed in modern times—that of Duhamel, and that of Bordenave. The former attributed to the swelling of the periosteum and medullary membrane, to their prolongation from one fragment to the other, and to their ossification, the consolidation of the fracture. He held that this re-union took place, at one time by means of a simple external ferrule, at another by means of a double ferrule, one enveloping the periphery of the fragment, the other penetrating the medullary canal, where it forms a kind of wedge, of greater or less length.

Bordenave established different principles. He admits that the union and consolidation of broken bone takes place by the same mechanism as the healing of soft parts: led, without doubt, to this idea by what happens when the fractured parts are exposed, he thought he could recognize the existence of cellular and vascular granulations between the fragments. According to him these granulations united and became solid by the deposition of phosphate of lime in their interior. These doctrines, more or less modified, were received down to our time, when in 1808, having undertaken to verify the ideas of Bordenave and Bichat, I was astonished to find nothing which justified them. I multiplied my researches and was led by numerous experiments to establish a theory partly founded on that of Duhamel, and which I taught in my course of pathological anatomy. Let us trace the most remarkable phenomena which we observe during the time that a fractured bone is becoming consolidated.

If the parts be examined between the first and tenth days, we find an extravasation of blood round the fragments, between them and in the medullary canal. The ecchymosis may extend to very distant parts. Inflammation and tumefaction to a considerable extent is developed at the irritated points. The fleshy fibres become confounded with the inflamed cellular tissue, and soon cease to be distinguishable from other parts. The periosteum becomes red and swollen, is softened and pours out a reddish serous fluid between it and the portions of bone which it covers. The medullary tissue becomes tumefied and inflamed, effacing by degrees the canal which the centre of the bone presents. The marrow becomes in some measure fleshy, and unites to that of the opposite side. If we examine what is going on with regard to the fragments, we find the clot which separates them to be absorbed in a few days, and replaced by a gelatinous secretion. From the fourth to the sixth day the surfaces of the fracture are covered with a reddish substance, of a downy appearance, but which is not always present. From the tenth to the twenty-fifth day the tumefaction of the soft parts becomes more solid; its adherence to the intermediate substance of the fragments appears every day more intimate; the muscles resume their wonted aspect and functions. The tumor, which I have called *tumeur du cal,* diminishes in extent, and separates from the surrounding parts; the tissue which composes it is homogeneous, like fibro-cartilage, and difficult to divide. If detached, it is found to consist of fibres parallel to the axis of the fractured bone. The swollen medullary membrane is transformed into fibro-cartilage, and progressively narrows the central cavity of the bone, till it finishes by wholly obliterating it.

In proportion as we advance in the examination of the formation of the callus, we observe other particulars: the process may go on to the twenty-fifth, fortieth, or even sixtieth day. In weakly subjects the work is not completed under three months. The lardaceous and fibrous mass which constitutes the "tumor of the callus," and which entirely envelopes the fragments, becomes by degrees cartilaginous.—Towards the end of the time, the fragments are included in the centre of solid ferrule, which adheres to them through the whole extent of the outer surface. Externally this ferrule is covered by thickened periosteum, which passes into that covering the sound portions of the bone. The cellular tissue in the neighborhood is still in a condensed state. The soft substance which was interposed between, in fragments, has now become more dense and more adherent to the extremities of the bone, but is yet far from uniting them in a perfect manner. The central peg continues to be prolonged towards the extremities, rapidly increases in consistence, and soon forms a very solid cylinder of bone. It is usually at this period that the apparatus is removed, but this callus is not yet to remain; consequently I have named it the "provisional callus," to point out that nature removes it to establish other means of union between the fragments.

From the third to the fifth, even to the sixth month, the tumor of the callus becomes gradually more compact, and the central portion undergoes the same transformation. The substance between the fragments acquires all the characters and consistence of compact bone, differing only in color. It is the transformation of this substance into bone that I have called the "definitive callus." In the concluding period of the formation of callus, the central portion becomes less dense; cells appear in its interior; it is converted into a reticular tissue, which itself finally disappears, and leaves the central canal of the bone perfectly free. The cells are then lined with a medullary membrane. After the establishment of the canal of the bone, it becomes continuous with the lining membrane which secretes the marrow. The external portion of the provisional callus also finishes by disappearing. It is to be understood that the different circumstances of fractures produce some slight varieties in those which attend the callus. Thus, when the fractured bones ride, the interior portion or peg is not found, and the same happens when the bone has no medullary cavity.

To recapitulate:—The re-union of bone generally offers the following phenomena: 1. Effusion of blood and viscid fluid. 2. Ecchymosis in the cellular tissue surrounding the extremities of the fracture. 3. The formation of a cartilaginous and bony ferrule externally, and of a kind of peg within. 4. Ossification of the substance interposed between the fragments. 5. Decrease of the tumor of the callus, and restoration of the medullary canal. The term of forty days, mentioned by many, is far from being sufficient; and where the fracture is oblique, or the bones ride, a much longer time is required.—*Boston Journal.*

26. *Gangrene of the mouth in Children.*—The following description of this terrible disease, which is certainly rare among us, we take from the Clinique of M. Guersent, physician to the hospital for children at Paris.

The peculiar subjects of this disease are children living in narrow and ill-ventilated situations, badly nourished, and whose constitution has been enfeebled by preceding maladies. According to some authors, its origin is very peculiar; the period of the invasion being marked by a small white spot, which soon becomes gangrenous and involves in its consequences the sphacelus of the parts adjacent. M. Guersent admits this to be often the case, but contends that it also frequently arises from simple inflammation of the gums. It may also arise from caries of the bones of the face in scrofulous subjects;—generally in this case the gangrene commences at the border of the fistulous opening, which transmits outwardly the remains of the suppurating bone. In whatever manner it appears, the march of the malady is generally the same: the tissues suddenly assume a greyish black appearance, and diffuse a gangrenous odor wholly different from the fetor of false membranes: the affected part swells, the cheek becomes tense and smooth, and in examining the interior of the mouth we easily see that the soft parts are reduced to a state of soft putrescence and that the teeth are loosened. This state may continue a greater or less length of time; the malady may remain stationary, or may continue its ravages in the buccal cavity without appearing outwardly; but generally at the end of five or six days the tension of the cheek augments, and a deep violet spot shows itself in the centre of the tumor. The epidermis softens and is easily removed at this point, which extends gradually, and sometimes finishes by carrying away the whole side of the face. The presence of the violet color indicates that all the parts, from the mucous to the exterior, are completely sphacelous. By degrees the eschars soften, fall, and discover the bones of the jaw, affected with necrosis and deprived of their teeth. Often the children remove those themselves, without appearing to experience the slightest pain. As those who are affected with gangrene of the mouth have almost always some visceral affection the fever is very decided through the whole disease. The absorption of the fetid ichor which inundates the gums and the interior of the mouth contributes to give it an adynamic character; the children are weakened by degrees; an abundant and fetid diarrhœa supervenes, and they finally fall victims to a true poisoning when the forces of nature do not suffice to free the system by the usual emunctories from the matters absorbed. Some children preserve their appetite to the last moment, and eat with voracity all the aliments presented them. Nothing can be more hideous than these repasts, in which putrid matters and even teeth are introduced into the stomach. Those children, however, whose appetites continue, usually resist the malady the longest time.

In the post-mortem examination, the œsophagus is ordinarily found filled with a blackish and very fetid secretion, the whole thickness of the cheek converted into eschars, and the bones of the face *necrosed.* These necroses, the extent of which is infinitely variable, present a special character which seems to distinguish them from all others.

The osseous tissue, struck with death, is black and dry: it would seem, says M. Guersent, as if the bone had contributed all its fluid portion to augment the putrid degeneration of the soft parts.

From the very first period the absence of false membranes will prevent our confounding gangrene of the mouth with the flaky stomatitis. As little can it be confounded with carbuncle, whose native situation and march are altogether different. Some varieties of pustula maligna approach it in some degree; but we may remark that this affection always commences with the skin, and attacks the deeper parts only consecutively; while gangrene of the mouth commences internally with the mucous membrane, and makes its ravages in the mouth itself before extending itself to the exterior.

*Treatment.*—Messrs. Baro, Jonard and Guersent consider the actual cautery as the most efficacious local therapeutic to avert the progress of the disease. The application ought to be repeated according to the intensity and extent of the disease. It is not well to have recourse to caustics, such as the butter of antimony and the acids, except in cases where the disease occupies the bottom of the cavity, so that it is impossible to apply the cautery. All these caustics are attended with the inconvenience of exciting an abundant flow of saliva, by which their action is exceedingly impaired. On the contrary, cauterizing may be carried to any depth we will, by submitting the point in question to the actual cautery.

The nitrate of silver has been highly praised by some practitioners; but the eschar which it produces is too superficial; and if sufficient of the salt be employed to destroy a certain thickness of tissue, there would be danger that the patients would swallow a portion of it.

Sometimes the whole effect of these cauterizations is nothing, and the disease continues its course; but in other cases the eschars are detached, the wounds clean, and the extensibility of the substance of the cheek is such that the small deformity which results from the cicatrization is not at all comparable with what would otherwise have existed.

In the treatment of this complaint, constitutional means ought to be insisted upon. As all the stages of it indicate a profound alteration of the fluids, M. Guersent advises the employment of tonics, to be modified according to the state of the organs diseased. The gargles ought always to be of a detersive or antiseptic nature; those composed of the decoction of cinchona and of chloride of soda are preferable.—*Boston Journal.*

## Figures and Tables

**Figure f1:**